# Display of whole proteins on inner and outer surfaces of grapevine fanleaf virus‐like particles

**DOI:** 10.1111/pbi.12582

**Published:** 2016-07-29

**Authors:** Lorène Belval, Caroline Hemmer, Claude Sauter, Catherine Reinbold, Jean‐Daniel Fauny, François Berthold, Léa Ackerer, Corinne Schmitt‐Keichinger, Olivier Lemaire, Gérard Demangeat, Christophe Ritzenthaler

**Affiliations:** ^1^ SVQVINRAUniversité de StrasbourgColmarFrance; ^2^ Institut de Biologie Moléculaire des Plantes CNRS‐UPR 2357associée à l'Université de StrasbourgCNRSStrasbourgFrance; ^3^ Institut de Biologie Moléculaire et Cellulaire du CNRSUPR 9002Architecture et Réactivité de l'ARNUniversité de StrasbourgStrasbourgFrance; ^4^ Institut Français de la Vigne et du VinDomaine de l'EspiguetteLe Grau‐du‐RoiFrance

**Keywords:** virus like particles, nanoparticles, virus, nepovirus, nanocarrier

## Abstract

Virus‐like particles (VLPs) derived from nonenveloped viruses result from the self‐assembly of capsid proteins (CPs). They generally show similar structural features to viral particles but are noninfectious and their inner cavity and outer surface can potentially be adapted to serve as nanocarriers of great biotechnological interest. While a VLP outer surface is generally amenable to chemical or genetic modifications, encaging a cargo within particles can be more complex and is often limited to small molecules or peptides. Examples where both inner cavity and outer surface have been used to simultaneously encapsulate and expose entire proteins remain scarce. Here, we describe the production of spherical VLPs exposing fluorescent proteins at either their outer surface or inner cavity as a result of the self‐assembly of a single genetically modified viral structural protein, the CP of grapevine fanleaf virus (GFLV). We found that the N‐ and C‐terminal ends of the GFLV CP allow the genetic fusion of proteins as large as 27 kDa and the plant‐based production of nucleic acid‐free VLPs. Remarkably, expression of N‐ or C‐terminal CP fusions resulted in the production of VLPs with recombinant proteins exposed to either the inner cavity or the outer surface, respectively, while coexpression of both fusion proteins led to the formation hybrid VLP, although rather inefficiently. Such properties are rather unique for a single viral structural protein and open new potential avenues for the design of safe and versatile nanocarriers, particularly for the targeted delivery of bioactive molecules.

## Introduction

From a structural and biotechnological standpoint, virions can be viewed as self‐assembled nanometre‐scale cages or viral nanoparticles (VNPs) containing the viral genome. Genetic engineering has allowed the development of nucleic acid‐free VNPs also named virus‐like particles (VLPs) that can be regarded as a subclass of VNPs. VNP‐based nanomaterials have been adapted through chemical reactions or genetic engineering to gain new functionalities. First, their external surfaces can be modified or decorated with other molecules, in which case VNPs can act as biocompatible nanocarriers for antigen presentation, immunomodulation, customized targeting, etc.*,* as exemplified by the plant‐infecting cowpea mosaic virus (CPMV, Chatterji *et al*., 2004; Lewis *et al*., 2006; Sainsbury *et al*., 2010; Steinmetz *et al*., 2011), tobacco mosaic virus (TMV, Alonso *et al*., 2013) or cowpea chlorotic mottle virus (CCMV, Suci *et al*., 2007). Second, as self‐assembled cages, the inner cavity of VNPs can be used to encapsulate or encage a variety of active molecules, including pharmaceuticals, image enhancers and nucleic acids (Arcangeli *et al*., 2014; Bruckman *et al*., 2013; Mueller *et al*., 2011; Shriver *et al*., 2013). By acting on both external surface and inner cavity, VNPs can be adapted theoretically at will and are therefore regarded as extremely versatile tools with great potentials in medicine, as enzyme nanocarriers or even as novel biomaterials (for review, see Reference Alonso *et al*., 2013; Cardinale *et al*., 2012; Pokorski and Steinmetz, 2010).

Despite the interesting traits offered by VNPs, the possibilities to modify them are often limited due to numerous constraints. For instance, whether derived from attenuated or killed animal viruses, bacteriophages or plant viruses, VNPs may contain a viral genome and may therefore be considered as hazardous infectious entities. In this respect, VLPs are highly advantageous over VNPs, because they are noninfectious and a multitude of viral structural proteins have been adapted to this end (for review see Reference Chen and Lai, 2013; Liu *et al*., 2012).

In general, while the external surface of VLPs can easily accommodate some modifications, the encapsulation capacity of VLPs is often restricted to only small molecules such as ions, oligonucleotides, pharmaceuticals or small peptides (for review, see Reference Glasgow and Tullman‐Ercek, 2014; Li and Wang, 2014; Saunders and Lomonossoff, 2013). To date, relatively few examples of encapsulation of foreign cargo proteins have been reported. These are limited to VLPs derived from viruses with spherical architecture, involves at least two separate protein components or nucleic acid linkers and is rather restrictive in stoichiometry of encapsulated proteins (for review, see Reference Lee, 2016; Lua, 2014; Sainsbury *et al*., 2014). To our knowledge, VLP made from a single structural protein compatible with the simultaneous inner and outer exposure of entire proteins via genetic fusions has not yet been described.

Grapevine fanleaf virus (GFLV) is the main causal agent of fanleaf degeneration, probably the most detrimental viral disease on the emblematic crop grapevine. GFLV belongs to the genus *Nepovirus* in the family Secoviridae (order *Picornavirales*) that also includes the *Comovirus* CPMV (Sanfaçon *et al*., 2009). GFLV, which has a bipartite positive‐sense RNA genome, is a nonenveloped icosahedral virus of approximately 30 nm in diameter, comprising 60 identical CP subunits of 56 kDa, arranged in a pseudo T = 3 symmetry, whose structure has been resolved at 2.7 Å resolution (Schellenberger *et al*., 2011b). Particles have been observed in transgenic plants expressing GFLV CP coding sequence, suggesting that the CP of GFLV is able to self‐assemble into VLPs (Barbier *et al*., 1997). Here, we show that the CP of GFLV is a highly versatile protein of biotechnological interest, compatible with the simultaneous encapsulation and exposure of large proteins through the genetic fusion to the CP N‐ or C‐terminal end.

## Results

### GFLV coat protein self‐assembles into virus‐like particles

**Figure 1:**
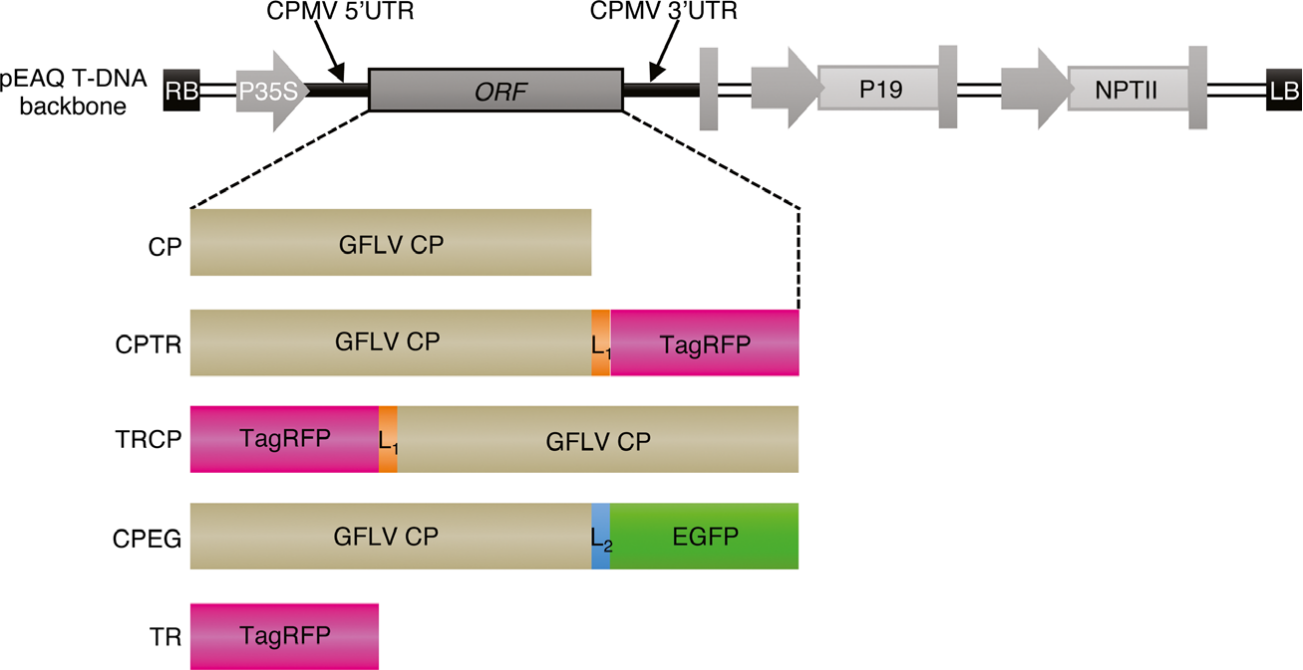
Schematic representation of pEAQ vector T‐DNA region and constructs derived thereof. The backbone of the T‐DNA region, extending from the right border (RB) to the left border (LB), is represented in grey shades. Open‐reading frame (ORF) of interest is flanked by sequences of cowpea mosaic virus untranslated regions (CPMV UTRs) under the control of cauliflower mosaic virus 35S promoter (P35S). Native GFLV CPs as well as its TR‐ and EG‐tagged variants (CPTR, TRCP, CPEG) or TagRFP alone (TR) were introduced into the pEAQ vector as schematically indicated. L1 corresponds to the 7‐amino‐acid Gly_3_‐Ser‐Gly_3_ linker sequence. L_2_ corresponds to the 15‐amino‐acid linker sequence resulting from Gateway recombination. Complete amino acid sequences of the expressed proteins are provided in Figure S1. P19: tombusvirus P19 silencing suppressor gene. NPTII: neomycin phosphotransferase II gene.

**Figure 2:**
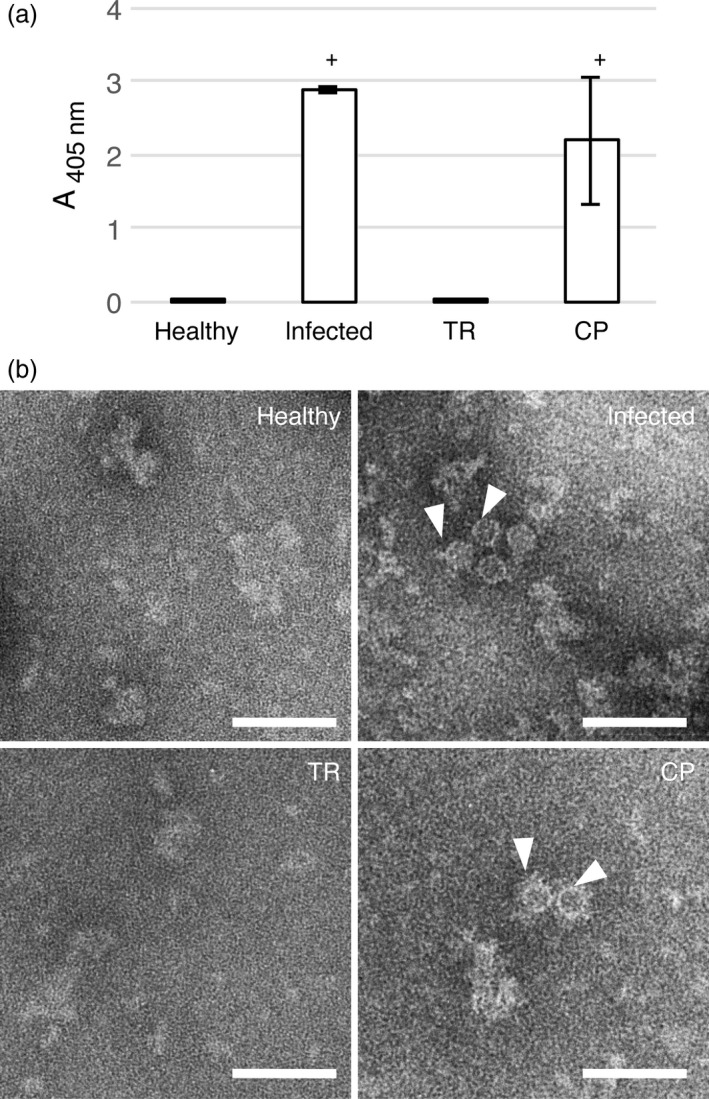
Transient expression of GFLV CP in *N. benthamiana* leaves leads to VLP production. (a) Expression of GFLV CP in *N. benthamiana* leaves at 7 dpa (TR and CP) or at 14 days of infection was determined by DAS‐ELISA using anti‐GFLV antibodies for detection. Bars represent the mean absorbance obtained with three different leaves for each condition. Error bars correspond to 95% confidence intervals. Samples were considered positive (+) when A_405 nm_ exceeded the healthy control sample mean value by at least a factor of three. (b) ISEM micrographs resulting from observations performed on the same extracts were then analysed by DAS‐ELISA. Particles of approximately 30 nm (arrowheads) were detected only in GFLV‐infected and CP‐expressing samples. Scale bars: 100 nm.

To address the ability of GFLV CP to produce VLPs *in planta,* the sequence encoding the CP from GFLV isolate F13 was introduced into the pEAQ‐*HT*‐DEST1 binary vector (Sainsbury *et al*., 2009) and used for transient expression in *Nicotiana benthamiana* leaves by agro‐infiltration (Figure 1). Samples were analysed by double‐antibody sandwich ELISA (DAS‐ELISA) at 7 days postagro‐infiltration (dpa) using GFLV‐F13‐infected *N. benthamiana* leaves at 14 days postinoculation as a positive control and pEAQ‐*HT*‐DEST1‐driven TagRFP (TR, Merzlyak *et al*., 2007) agro‐infiltrated leaves at 7 dpa and leaves from healthy plants as negative controls. A strong positive signal was detected in CP‐expressing and GFLV‐infected samples but not in extracts from TR‐infiltrated or healthy leaf material (Figure 2a). To test the ability of transiently expressed CP to self‐assemble into VLPs, the same leaf extracts were further analysed by transmission electron microscopy (TEM) after immunocapture with GFLV‐specific polyclonal antibodies (see experimental procedures). Observation of negatively stained material (Figure 2b) revealed the presence of icosahedral particles of about 30 nm in diameter in CP‐expressing samples but not in TR‐expressing or healthy negative controls (Figure 2b). Although not very abundant on grids, CP‐derived icosahedral particles were very similar to GFLV particles observed under equivalent conditions (Figure 2b). This indicates that GFLV CP is able to self‐assemble into VLPs upon transient expression in *N. benthamiana*.

### N‐ and C‐terminal CP fusion proteins assemble into VLPs

**Figure 3:**
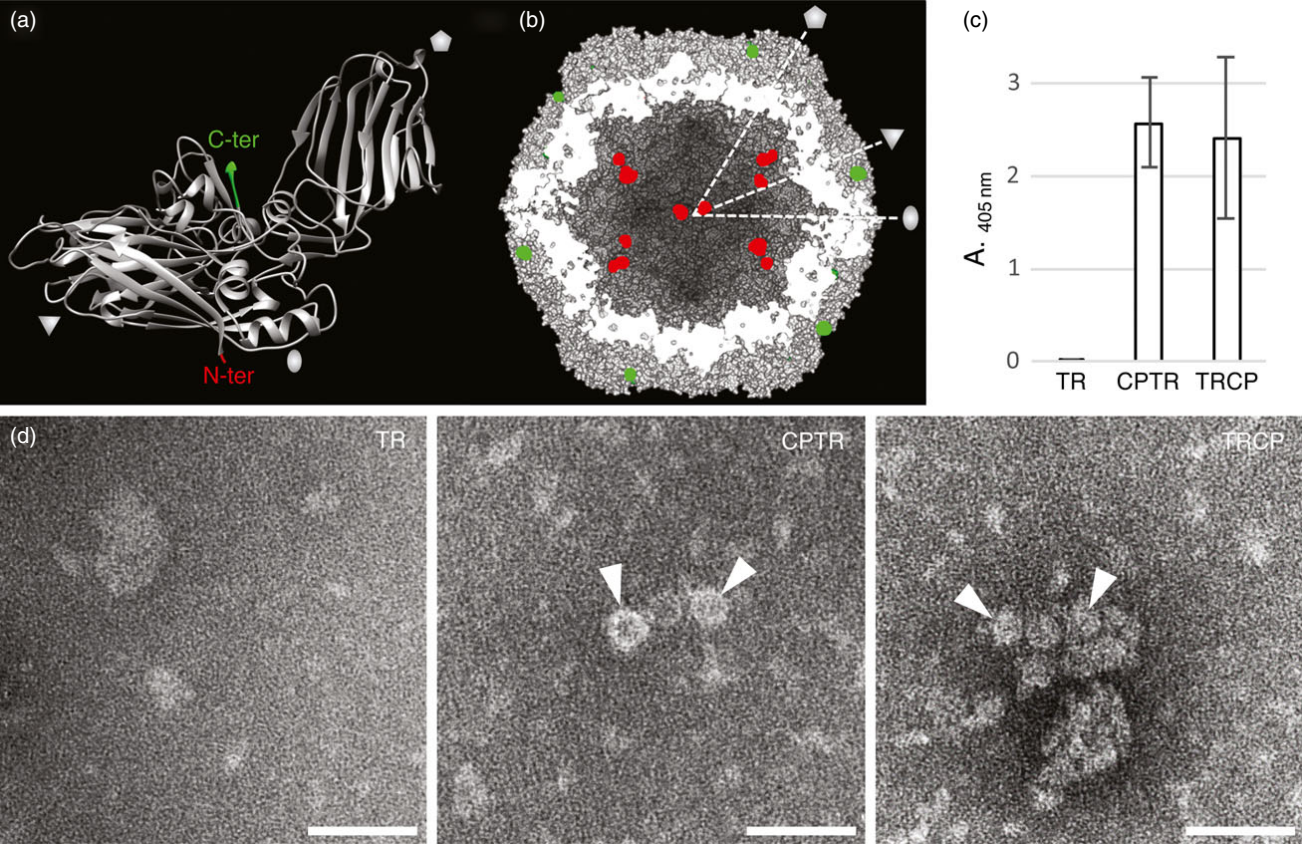
Fusion of TagRFP to the N‐ or C‐terminal end of GFLV CP is compatible with VLP formation. (a) Ribbon diagram view of a GFLV CP subunit and (b) surface‐shaded cross section of a particle according to the 3 Å resolution crystal structure (PDB code 4V5T, (Schellenberger *et al*., 2011b). Positions of the CP N‐ and C‐termini are indicated in red and green, respectively. The pentagon, triangle and oval symbolize the fivefold, threefold and twofold icosahedral symmetry axes, respectively. Cross‐sectioned residues appear in white. (c) Detection of GFLV CP in TR‐, CPTR‐ or TRCP‐expressing *N. benthamiana* crude leaf extracts at 7 dpa. Bars represent the mean absorbance obtained with three different leaves for each condition. Error bars correspond to 95% confidence intervals. (d) ISEM micrographs resulting from observations performed on the same extracts than analysed by DAS‐ELISA. Arrowheads point to VLPs trapped by anti‐GFLV antibodies in CPTR‐ and TRCP‐expressing leaf extracts. Scale bars: 100 nm.

Analysis of the GFLV atomic structure (Schellenberger *et al*., 2011b) reveals that the GFLV CP amino‐terminal residue Gly_1_ and the three carboxy‐terminal residues Phe_502_, Pro_503_ and Val_504_ do not contribute to the quaternary interactions of the virus capsid and are exposed at the inner and outer surfaces of the GFLV particle, respectively (Figure 3a and 3b). In this respect, both extremities could be expected compatible with the addition of extra residues without interfering with the capacity of the CP to form a capsid. To test this hypothesis, N‐ or C‐terminal CP fusions to TR were produced and, respectively, named TRCP and CPTR hereafter (Figure 1). Both fusions included a Gly_3_‐Ser‐Gly_3_ linker peptide (Figure 1 and Figure S1) to maintain flexibility between the CP and TR domains (Zilian and Maiss, 2011) and were transiently expressed in *N. benthamiana* leaves. Samples were analysed by epifluorescence macroscopy for TR expression at 5 dpa (Figure S2), and 2 days later by DAS‐ELISA for CP expression (Figure 3c) and TEM for VLPs (Figure 3d). While TR fluorescence was observed in all samples (Figure S2) suggesting the expression of the different proteins, CP was detected only in CPTR and TRCP crude extracts by DAS‐ELISA (Figure 3c), which correlated with the presence of VLPs in TEM (Figure 3d). These results suggest that GFLV CP retains its capacity to form VLPs upon fusion of its N‐ or C‐terminal end to TR.

**Figure 4:**
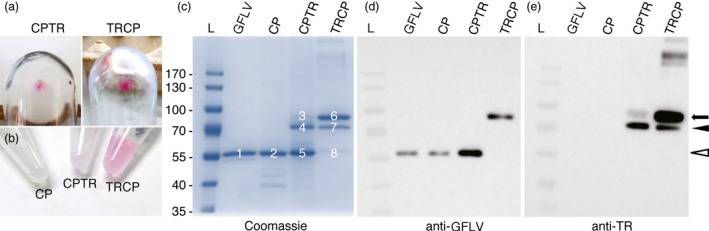
Recombinant VLPs can be purified from CP‐, CPTR‐ and TRCP‐expressing leaves. (a) Pink pellets resulting from CPTR (left panel) and TRCP (right panel) purifications after final ultracentrifugation. (b) Purified CP, CPTR and TRCP in solution. Note the pink colour of CPTR and TRCP samples. (c) Coomassie blue‐stained gel of GFLV‐, CP‐, CPTR‐ and TRCP‐purified particles after SDS‐PAGE. Six micrograms of GFLV particles equivalent was separated in each lane. Major bands in the gel are numbered from 1 to 8. (d and e) Corresponding Western blotting analyses of GFLV, CP, CPTR and TRCP samples using anti‐GFLV (d) or anti‐TR antibodies (e). About 0.05 μg of GFLV particles equivalent was used in each lane. White arrowhead indicates bands with expected size for CP. Arrow points to expected size for full‐length TRCP or CPTR fusions. Black arrowhead points to major TRCP or CPTR truncated products. L: molecular mass markers. Masses (kDa) are indicated in the left.

To confirm our results and to gain insights into the biochemical properties of VLPs, large‐scale production of VLPs in *N. benthamiana* leaves was carried out followed by their purification using standard GFLV purification procedure in the absence of protease inhibitors (see experimental procedures). In parallel, GFLV was purified from infected *Chenopodium quinoa* leaves. After linear sucrose gradient, a pink band was observed in the TRCP gradient (Figure 3Sa). Two millilitres of sucrose gradient fractions was collected and those enriched in VLPs identified by semiquantitative DAS‐ELISA. While *bona fide* GFLV particles sedimented essentially towards the bottom of the gradient in fractions 8—10, CP‐, CPTR‐ and TRCP‐derived particles distributed to the lighter fractions 3—5, 4—6 and 6—8, respectively (Figure S3b), well in agreement with a previous report indicating that empty GFLV particles show lower sedimentation coefficient than RNA‐containing virions (Quacquarelli *et al*., 1976). Enriched fractions were further pooled and processed for final concentration by ultracentrifugation. Remarkably, pink pellets were observed in both TRCP and CPTR samples (Figure 4a). The final concentration of purified material (Figure 4b) was determined by quantitative DAS‐ELISA using purified GFLV as a standard. Determined yields ranged from 386 to 445 μg GFLV particles equivalent per kg of fresh leaves for the three VLP types, which is in the same order of magnitude as GFLV purification yields from infected *N. benthamiana* (Schellenberger *et al*., 2011a).

**Figure 5:**
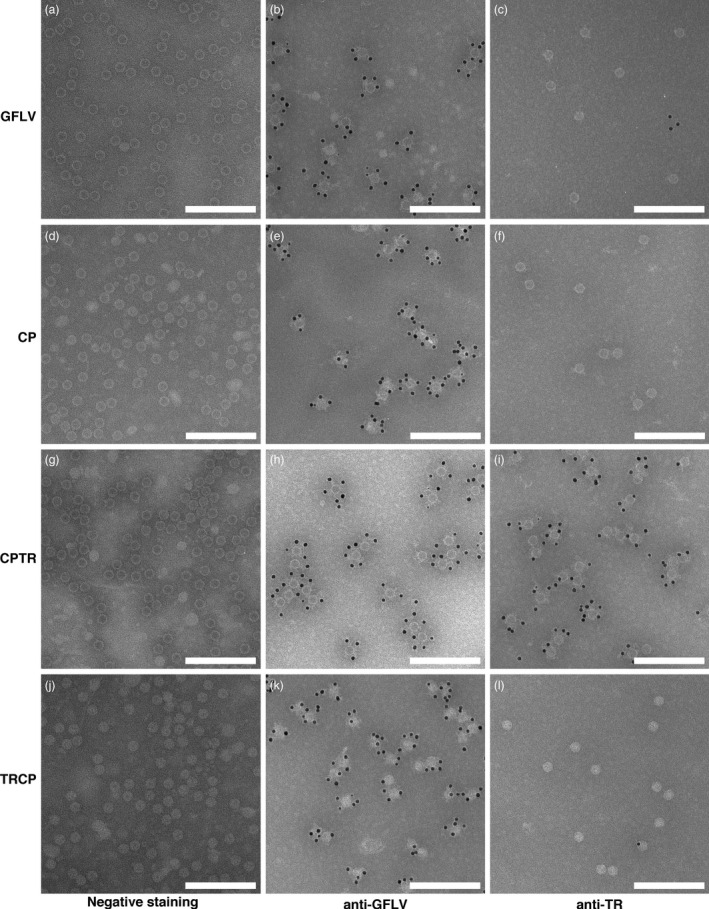
Proteins fused to the N‐ or C‐terminal end of GFLV CP are encaged or exposed at the outer surface of VLPs, respectively. Electron micrographs of purified GFLV (a, b, c), CP VLPs (d, e, f), CPTR VLPs (g, h, i) and TRCP VLPs (j, k, l). Samples were processed for negative staining only (a, d, g, j) or for ISEM using anti‐GFLV (b, e, h, k) or anti‐TR (c, f, i, l) antibodies and antibodies conjugated to 10‐nm colloidal gold particles for labelling. Scale bars: 200 nm.

**Figure 6:**
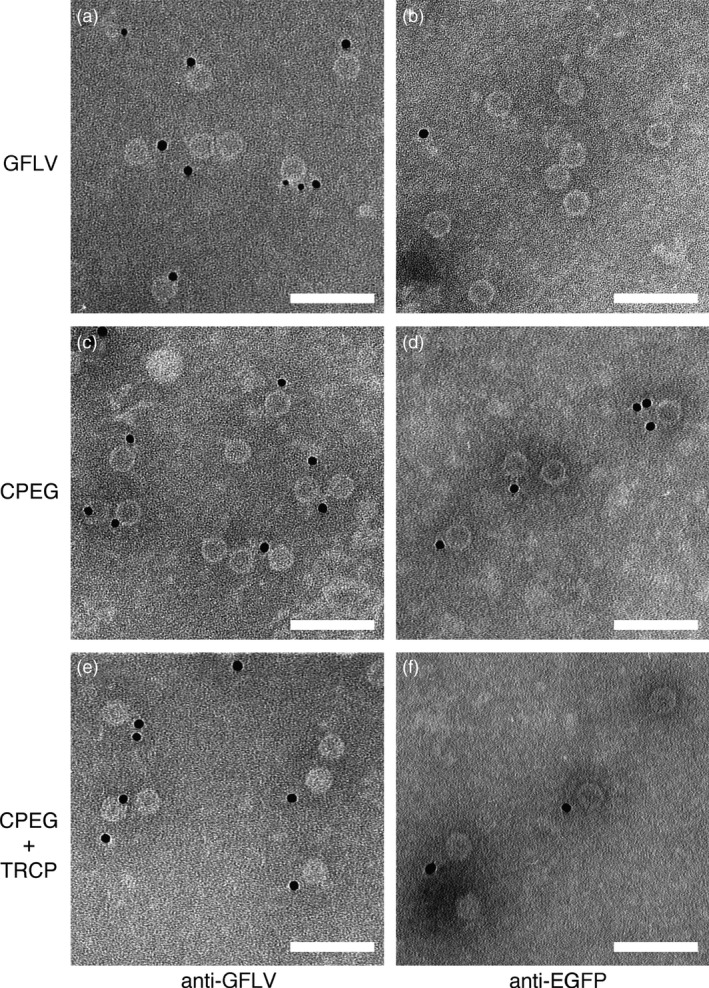
VLPs can be purified from *N. benthamiana* leaves coexpressing CPEG and TRCP. Transmission electron micrographs of purified GFLV (a, b), CPEG VLPs (c, d) and CPEG + TRCP VLPs (e, f) after immunogold labelling. Samples were processed for ISEM using anti‐GFLV (a, c, e) or anti‐EGFP (b, d, f) antibodies and secondary antibodies conjugated to 10‐nm colloidal gold for decoration. Scale bars: 100 nm.

To assess their quality and purity, purified samples were analysed by Coomassie blue staining after SDS‐PAGE (Figure 4c), immunoblotting using anti‐GFLV or anti‐TR antibodies (Figure 4d and 4e) and mass spectrometry (Figure S4). For Coomassie blue staining, 6 mg of GFLV particles equivalent of each sample was loaded on SDS‐denaturing gel. In line with the purification of VLPs, one major protein of about 57 kDa co‐migrating with the CP of GFLV (calculated mass 56 kDa) was present in purified samples from CP‐expressing leaves (Figure 4c, bands 1 and 2). For CPTR and TRCP samples, profiles were more complex with three major proteins of approximate molecular masses of 87, 73 and 57 kDa being detected in both samples (Figure 4c, bands 3—5 for CPTR and 6—8 for TRCP), but in inverse proportions. For TRCP, the largest product was the most abundant and the smallest the least abundant (approximately 69%, 24% and 7% respective abundance), whereas for CPTR the proportions were as follows: 2% for band 3, 35% for band 4 and 63% for band 5. Upon immunoblotting with anti‐GFLV antibodies, the shortest product present in the CPTR sample (Figure 4c, b and 5) was clearly detected (Figure 4d), strongly suggesting that band 5 corresponds to the CP of GFLV and probably represents a processing product of CPTR. In the TRCP sample, the largest product (Figure 4c, b and 6) immunoreacted clearly with anti‐GFLV antibodies (Figure 4d). Considering this band is about the expected size of TRCP (calculated mass 82.8 kDa), our results suggest that the full‐length TRCP is the principal protein present in the purified TRCP sample. Accordingly, band 6 gave also a strong signal upon immunodetection with anti‐TR antibodies (Figure 4e). Anti‐TR antibodies immunoreacted also but weakly with the largest product present in the CPTR sample (band 3) and with the 73 kDa truncated products observed in CPTR and TRCP samples (Figure 4e, bands 4 and 7). Our results suggest that TRCP and CPTR have the capacity to self‐assemble into VLPs *in planta*. They also reveal clear differences in the capacity of these VLPs to accommodate fusion proteins with TRCP‐derived VLPs being far less prone to degradation or truncation than CPTR‐derived ones.

To gain further insights into the composition of the purified products, Coomassie‐stained bands were subjected to mass spectrometry leading to the identification of peptides covering nearly the entire CP for each band analysed (Figure 4c and Figure S4). Peptides corresponding to TR were only observed for bands 3, 4, 6 and 7. Nearly full coverage of the CPTR or TRCP proteins was strictly restricted to bands 3 and 6. The 73 kDa products corresponding to bands 4 and 7 showed only partial coverage of the TR and thus represent a truncated version of CPTR or TRCP, possibly due to proteolytic degradation during the purification process performed without protease inhibitors. Our results demonstrate that the CPTR or TRCP full‐length chimeric proteins can be purified following standard virus purification procedures likely reflecting their capacity to self‐assemble into VLPs. They also reveal that the CPTR fusion is more labile than TRCP, possibly as a consequence of the predicted orientation of TR towards the VLP inner or outer surface.

### N‐ and C‐terminal CP fusions are oriented towards the interior and exterior of VLPs, respectively

To confirm the presence of VLPs and the different orientation shown by TR in CPTR and TRCP samples, negative staining and immunosorbent electron microscopy (ISEM) analyses were performed. As expected, negative staining revealed the presence of numerous VLPs in all samples (Figure 5d, 5g and 5j) that clearly resemble GFLV particles (Figure 5a). Under these conditions, the interiors of CP and CPTR particles appeared electron dense (Figure 5d and 5g) similar to those of GFLV particles (Figure 5a), whereas those of TRCP particles appeared rather electron lucent (Figure 5j), likely reflecting the increased inner density of particles and decreased penetrability to heavy metals linked to the orientation of TR inside TRCP VLPs. To verify the topology of VLPs, decoration assays were performed with anti‐GFLV (Figure 5b, 5e, 5h and 5k) or anti‐TR antibodies (Figure 5c, 5f, 5i and 5l). While all purified particles reacted to anti‐GFLV antibodies as expected (Figure 5b, 5e, 5h and 5k), only CPTR particles were decorated with anti‐TR antibodies (Figure 5i), despite the significantly greater proportion of full‐length protein present in TRCP versus CPTR particles (Figure 4). The observed differences in accessibility to TR antibodies confirm the exposure of TR at the outer surface of CPTR‐derived VLPs and the encaging of TR inside the particles in TRCP‐derived VLPs. Perhaps most importantly, our results also clearly show that GFLV CP can accommodate the fusion of foreign proteins as large as fluorescent proteins (FP) that represent approximately half the mass of the CP without losing its capacity to self‐assemble into VLPs.

### Hybrid VLPs can be produced

**Figure 7:**
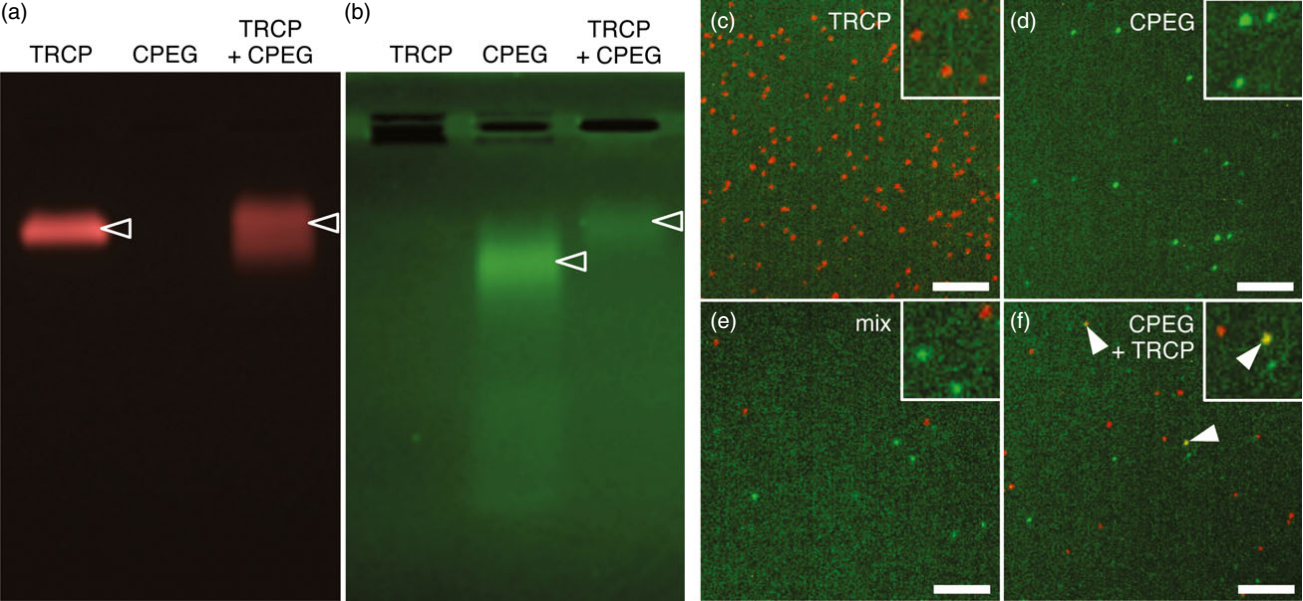
Hybrid VLPs are produced upon coexpression of CPEG and TRCP. (a, b) Fluorescence imaging of TRCP, CPEG or CPEG + TRCP VLPs separated by native agarose gel electrophoresis. Imaging was performed sequentially using a G:box imaging system, first at λ_ex_480—540 nm for excitation and λ_em_590—660 nm for emission to detect TR (a), then at λ_ex_450—485 nm Schellenberger *et al*., 2011b and λ_em_510—540 nm to detect EG (b). Fluorescent VLPs in the gel are indicated by empty arrowheads. (c to f) Single‐particle microscopy images of purified TRCP VLPs (c), CPEG VLPs (d), mixed TRCP and CPEG VLPs at 1 : 1 ratio (e) and coexpressed CPEG + TRCP VLPs (f). Epifluorescence imaging was performed sequentially at λ_ex_455—495 nm to λ_em_505—555 nm to detect EG and at λ_ex_532.5—557.5 to λ_em_570—640 nm to detect TR. White arrowheads point at hybrid VLPs. Scale bars: 5 μm.

In view of our results, we next examined the capacity of GFLV CP to form hybrid VLPs upon coexpression of N‐ and C‐terminal CP fusions. To do so, EGFP (named EG hereafter) was fused to the CP C‐terminus as indicated in Figure 1 (construct CPEG). As before, agro‐infiltrated *N. benthamiana* leaves were used for expression assays and purification of VLPs. CPEG‐only‐expressing leaves were used as negative control and compared to leaves coexpressing CPEG and TRCP (CPEG + TRCP). In compliance with our previous results, CPEG VLPs could be purified and located to the same linear sucrose gradient fractions as CPTR VLPs (Figure S3b). Coexpressed CPEG and TRCP also enabled the purification of DAS‐ELISA immunoreactive material cosedimenting with CPEG VLPs (Figure S3b). ISEM analysis confirmed the presence of VLPs in both CPEG and CPEG + TRCP samples that clearly immunoreacted with both anti‐GFLV and anti‐EG antibodies (Figure 6), well in agreement with the predicted exposure of EG towards the external surface of VLPs. Considering TR is inaccessible to antibodies in ISEM upon fusion to the CP N‐terminus, we further assessed the presence of EG and TR by fluorescence imaging of VLPs separated by native agarose gel electrophoresis (Figure 7). Under such conditions, distinct bands with specific migration profiles were detected for TRCP VLPs (Figure 7a), CPEG VLPs (Figure 7b) and CPEG + TRCP VLPs (Figure 7a and b) that emitted in both green and red channels as expected for hybrid particles (Figure 7a and 7b).

To confirm the production of *bona fide* hybrid VLPs and hence the presence of particles that emit simultaneously in green and red and should therefore appear yellow, purified samples were further processed for single‐particle microscopy. In this manner, TRCP VLPs appeared as numerous individual red spots (Figure 7c). Similarly, distinct green spots likely corresponding to CPEG VLPs were also detected but in lower density (Figure 7d), likely reflecting the low abundance of full‐length protein in purified CPEG VLPs samples. Importantly, the observation of a mix of separately purified CPEG and TRCP VLPs revealed individual red‐ or green‐only VLPs (Figure 7e). Although not abundant probably as a consequence of proteolysis of EG molecules on the outer surface of VLPs, yellow spots likely corresponding to hybrid VLPs were detected only in CPEG + TRCP‐derived samples (Figure 7f). Altogether, our results demonstrate that GFLV CP is compatible with the production of hybrid VLPs in which foreign proteins as large as FPs can be exposed at the outer surface and encaged inside individual VLPs when N‐ and C‐terminal fusions to CP are coexpressed.

### GFLV CP‐derived VLPs are nucleic acid free

**Figure 8:**
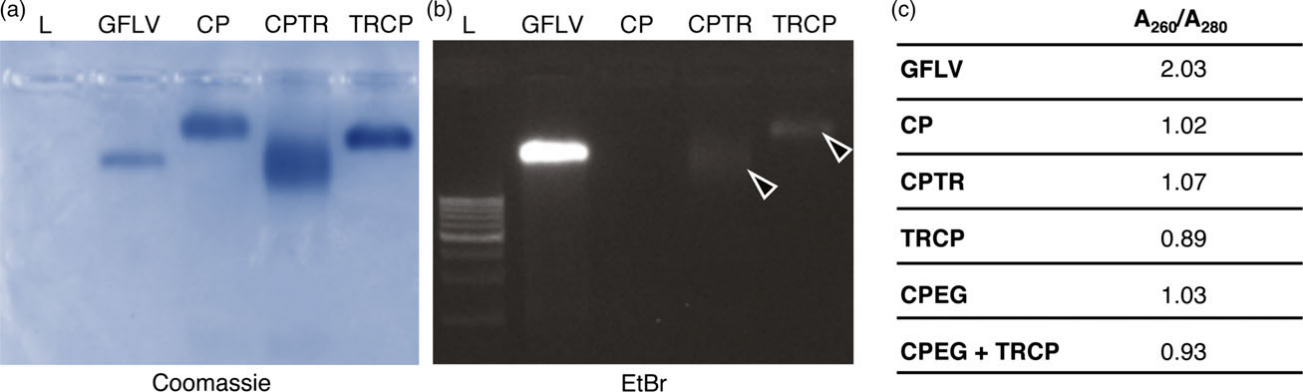
Purified VLPs are nucleic acid free. (a and b) Purified GFLV and CP, CPTR and TRCP VLPs separated by native agarose gel electrophoresis after Coomassie blue (a) and ethidium bromide staining (b). Arrowheads point to bands corresponding to crosstalk. (c) A_260_/A_280_ ratio of purified particles.

To examine the composition of VLPs, native agarose gel electrophoresis was performed and gel‐stained with Coomassie blue or ethidium bromide for protein (Figure 8a) or nucleic acid content (Figure 8b). Upon protein staining, the migration profiles of CP, CPTR and TRCP VLPs as well as purified GFLV differed significantly, probably as a consequence of differences in net charge, density and mass of the various particles. Under UV illumination, a clear signal was observed with purified virus but not with CP‐derived VLPs (Figure 8a). We attributed the faint bands observed with CPTR and TRCP VLPs (Figure 8b, arrowheads) to the slight TR excitation under UV light (Merzlyak *et al*., 2007) and the use of filters unable to fully discriminate TR and nucleic acid spectra rather than to the presence of nucleic acids. Upon spectrophotometer analysis, only purified virus led to high A_260/280_ values (>1.6) indicative of the presence of nucleic acids, whereas those measured for the different VLPs ranged from 0.89 to 1.07 (Figure 8c). Our results suggest that VLPs are, within the limits of our detection methods, nucleic acid free. Similar results were obtained with CPMV‐derived VLPs (Hesketh *et al*., 2015; Saunders *et al*., 2009).

## Discussion

We have shown that GFLV CP when expressed transiently in plants is able to form VLPs that can be purified using standard nepovirus purification procedures. Such VLPs look similar to native GFLV particles and appear nucleic acid free. Altogether, our results indicate that GFLV CP has the capacity to self‐assemble into VLPs in an RNA‐independent manner as already suggested by the presence of empty GFLV particles in samples from infected plants (Quacquarelli *et al*., 1976) and in CP‐expressing transgenic plants (Barbier *et al*., 1997). This capacity is probably a general feature of nepovirus‐encoded CPs considering empty particles are commonly observed upon the expression of nepoviral CPs in transgenic plants or insect cells (Barbier *et al*., 1997; Bertioli *et al*., 1991; Seitsonen *et al*., 2008; Singh *et al*., 1995) or upon virus purification (Lai‐Kee‐Him *et al*., 2013). Most remarkable is the ability of GFLV CP to maintain its capacity to self‐assemble into VLPs upon genetic fusion to proteins as large as FP that represent about half the size of the CP (256 residues including linker sequence for EG vs 504 for CP).

While CPs of numerous viruses accommodate genetic fusions and retain their ability to self‐assemble into VLPs, those are commonly restricted to peptides as exemplified with simian virus 40 (Takahashi *et al*., 2008), bacteriophage P22 (Servid *et al*., 2013) or CCMV (Brumfield *et al*., 2004; Hassani‐Mehraban *et al*., 2015) rather than full‐length proteins. Another common limitation observed with VLPs is linked to sites of insertion of foreign proteins not necessarily located at the extremities but often exposed within loops of the CP, as it is the case for hepatitis B virus (Kratz *et al*., 1999; Peyret *et al*., 2015) or CPMV (Porta *et al*., 2003), requiring the inserted proteins to be fused to both their extremities. From this viewpoint, GFLV CP appears rather special among the realm of VLP‐compatible CPs from animal, bacterial and plant viruses.

Certainly the most remarkable of findings is the differential orientation of the foreign proteins upon fusion to the N‐ or C‐terminal end of GFLV CP, resulting in either encaging or exposure of FP to the particle outer surface, respectively. While exposure of proteins of interest at the VLPs outer surface is commonly achieved, encaging of proteins is less frequent and currently only demonstrated for few icosahedral viruses such as CCMV in plant (Minten *et al*., 2009) or the well‐established example of human papillomavirus (Schiller and Lowy, 2012). To our best knowledge, identification of a single CP having the capacity to (i) self‐assemble into nucleic acid‐free VLPs, (ii) accommodate N‐ and C‐terminal genetic fusions and (iii) expose and encage large recombinant proteins is unique and highlights the versatility of the CP of GFLV as a nanocarrier. Despite the low primary sequence conservation among CP of nepoviruses (Sanfaçon *et al*., 2009), it is likely that the properties established here for the CP of GLFV extend to most if not all CPs from the genus *Nepovirus*. Hence, the reported atomic structures of tobacco ringspot virus (Chandrasekar and Johnson, 1998) and blackcurrant reversion virus (Seitsonen *et al*., 2008) and the backbone model of arabis mosaic virus (Lai‐Kee‐Him *et al*., 2013) all show CP N‐terminal ends facing the interior of the particles and CP C‐terminal residues exposed at the capsid outer surface.

GFLV atomic structure (Figure 3a, b; Schellenberger *et al*., 2011b) reveals that the N‐ and C‐terminal ends of all 60 CP subunits are surface accessible and should therefore accommodate fusion to proteins, at least theoretically. This seems in disagreement with our experimental data, in particular those concerning proteins exposed at the outer surface of VLPs that represent only a minor proportion in purified particles (Figure 4). The CP of tomato black ring virus (TBRV) was shown to undergo proteolytic processing resulting in the loss of the 9 C‐terminal amino acids (Demangeat *et al*., 1992). Although the CP of GFLV does not seem to undergo similar processing (Schellenberger *et al*., 2011b), the addition of residues may promote the cleavage of additional C‐terminal amino acids as already reported for CMPV‐derived VLP (Montague *et al*., 2011). Flexibility provided by the linker peptide may also contribute to increased fragility of the exposed recombinant proteins and their truncation. Probably the most likely explanations reside in the suboptimal transient expression system used and the purification procedure performed in the absence of protease inhibitors. This could clearly also account for the discrepancy in the proportion of full‐length recombinant proteins vs truncated products present in purified VLPs (Figure 4) as FPs encaged into VLPs are likely to be less prone to proteolysis than those exposed at the outer surface. Future work will aim at optimizing the VLP production system before multiple biotechnological applications can be foreseen. This includes reducing protein truncation in particular of outer surface‐exposed proteins which remain highly sensitive to proteolysis, improving VLPs purification yield and exploring whether a GFLV CP with both ends fused to foreign proteins still forms VLPs.

Finally, GFLV CP‐derived VLPs may also provide new perspectives to explore fundamental mechanisms such as tubule‐guided movement in plants (Amari *et al*., 2010; Ritzenthaler and Hofmann, 2007) and virus transmission from plant to plant by nematode vectors (Marmonier *et al*., 2010; Schellenberger *et al*., 2010). Indeed, the production of VLPs *in planta* in which FPs are encaged should prove to be a unique and powerful tool to track particles by fluorescence microscopy approaches, in particular, during intracellular and cell‐to‐cell movement of virus through plasmodesmata and also during the nematode feeding process.

## Experimental procedures

### Construction of binary plasmids

Coding sequences for GFLV‐F13 CP, TagRFP and EGFP were amplified by PCR using Phusion high‐fidelity DNA polymerase according to the manufacturer's instructions (New England Biolabs, Ipswich, MA; Thermo Fisher Scientific, Villebon sur Yvette, France) using pVec_Acc65I_2ABC (Schellenberger *et al*., 2010), pTagRFP‐C (Evrogen, Moscow, Russia) and pEGFP‐N1 (Clontech, Palo Alto, CA) as templates, respectively. The translational fusions TRCP and CPTR, corresponding, respectively, to N‐ or C‐terminal fusions of GFLV CP with TagRFP, were obtained by overlapping PCRs (Ho *et al*., 1989) using above‐described PCR products as templates and overlapping primers encoding the Gly_3_‐Ser‐Gly_3_ peptide linker sequence. The *att*B‐flanked CP, TR, CPTR and TRCP PCR products were cloned by Gateway recombination into the pDONR/Zeo donor vector (Invitrogen, Carlsbad,CA) and further recombined into the pEAQ‐*HT*‐DEST1 binary plasmid (Sainsbury *et al*., 2009). For CPEG, in which the C‐terminus of the CP is fused to EG, a pDONR/Zeo entry vector containing the CP coding sequence devoid of stop codon was used for recombination in a homemade Gateway expression vector deriving from the pEAQ‐*HT*‐DEST1 (Sainsbury *et al*., 2009) vector by the introduction of the EG encoding sequence downstream of the *att*R2 recombination site. Recombination resulted in the introduction of the DPAFLYKVVRSFGPA linker peptide between CP C‐terminal residue and EG (Figure 1 and Figure S1). All the primers used for cloning are available upon request.

### Plant material, virus infection and VLP production

*C. quinoa* and *N. benthamiana* plants were grown in chambers at 22/18 °C (day/night). GFLV infectious crude sap derived from pMV13 + pVec_Acc65I_2ABC‐infected material (Schellenberger *et al*., 2010) was used to mechanically inoculate three‐week‐old *C. quinoa*. Plants were harvested 14 days postinoculation and used for virus purification. For mechanical inoculations of *N. benthamiana,* three‐week‐old plants were inoculated with 300 ng of purified GFLV per plant. VLPs were produced by transient expression via agro‐infiltration of *N. benthamiana* leaves. Binary plasmids were introduced by electroporation into *Agrobacterium tumefaciens* strain GV3101 (pMP90). Cultures were grown to stable phase in Luria‐Bertani media with appropriate antibiotics, pelleted and resuspended in sterile water, alone or in a 1 : 1 ratio for coexpression, to a final absorbance of 0.5 at 600 nm. Suspensions were infiltrated into four‐week‐old *N. benthamiana* leaves with 2‐mL plastic syringes. Healthy, infected and agro‐infiltrated *N. benthamiana* plants were maintained in a growth chamber set at 14‐h light/10‐h dark photoperiod (4800 lx) with a temperature setting of 21/18 °C (day/night) for 7 days before leaf harvesting.

### Imaging of agro‐infiltrated leaves

FP visualization was realized at 5 dpa. Leaves were imaged with an AxioZoom V16 macroscope (Zeiss, Oberkochen, Germany) using 450‐ to 490‐nm/500‐ to 550‐nm excitation/emission wavelength filters for EG and of 538—562 nm/570—640 nm for TagRFP visualization. Images were processed using ImageJ (Schneider *et al*., 2012) and GNU Image Manipulation Program (GIMP, www.gimp.org).

### DAS‐ELISA

Healthy, infected and agro‐infiltrated leaves were ground at 1 : 5 w/v ratio in HEPES 100 mm pH 8, and saps were clarified for 5 min at 3000 ***g***. GFLV or VLP detection was performed using a commercial DAS‐ELISA kit (Bioreba, Reinach, Switzerland) according to the manufacturer's instructions. Briefly, plates were coated with polyclonal anti‐GFLV antibodies diluted at a 1 : 1000 in coating buffer, incubated with clarified extracts before the addition of anti‐GFLV monoclonal antibodies coupled to alkaline phosphatase at a 1 : 1000 dilution in conjugate buffer. Detection was realized using *para*‐nitrophenylphosphate and absorbance at 405 nm measured with a Titertek Multiskan MCC/340 reader (Labsystems, Helsinki, Finland). Samples were considered positive when the absorbance values exceeded the control samples by at least a factor of three.

### Negative staining, immunocapture and immunosorbent electron microscopy

Healthy, infected and agro‐infiltrated leaves were ground in HEPES 100 mm pH 8, and saps were clarified by centrifugation at 3000 ***g*** for 5 min and processed for negative staining, immunocapture or ISEM. Negative staining was performed on 300 mesh nickel grids covered with carbon‐coated Formvar (Electron Microscopy Science, Hatfield, PA) by incubation for 90 s with a 1% (w/v) ammonium molybdate solution. For immunocapture, grids were coated with polyclonal anti‐GFLV antibodies (Bioreba) at a 1 : 100 dilution, incubated with plant saps for 2 h at 4 °C, washed in HEPES 25 mm pH 8 buffer and finally processed for negative staining. For ISEM, grids were coated with in‐house monoclonal antibodies against GFLV at 0.05 mg/mL and incubated with VLPs for 1 h at room temperature. After blocking with 2% w/v BSA, 10% v/v normal goat serum, 0.05% Triton‐X100 in 22.5 mm HEPES pH 8, grids were further incubated with either anti‐GFLV (Bioreba) at a 1 : 100 dilution or anti‐TR polyclonal antibodies (Evrogen) at 0.01 mg/mL for 1 h at room temperature. Immunogold labelling was performed using anti‐rabbit antibodies conjugated to 10‐nm colloidal gold particles at 1 : 50 dilution (British Biocell International). Washes with HEPES 25 mm pH 8 were performed between all steps. ISEM was performed in a similar manner except that anti‐GFLV polyclonal antibodies (Bioreba) were used for capture and either a mix of anti‐GFLV monoclonal antibodies (Gaire *et al*., 1999) or anti‐EGFP monoclonal antibodies (Roche Diagnostics GmbH, Mannheim, Germany) employed for detection. Finally, immunogold labelling was performed using anti‐mouse antibodies conjugated with 10‐nm colloidal gold particles (British Biocell International). Observations were realized using a Philips EM208 transmission electron microscope. Film‐based photographs were acquired onto Kodak Electron Image Films SO‐163 (Electron Microscopy Science) and developed. Micrographs were scanned and images were processed using GNU Image Manipulation Program (GIMP, www.gimp.org).

### GFLV CP structure representation and analysis

CP subunit and capsid representations were made using the previously 3 Å resolved GFLV‐F13 atomic structure (PDB ID: 4V5T, (Schellenberger *et al*., 2011b) using the UCSF Chimera package (Pettersen *et al*., 2004). The CP subunit ends accessibility data were obtained using VIPERdb (Carrillo‐Tripp *et al*., 2009).

### Virus and VLP purification

GFLV was purified from *C. quinoa*‐infected plants according to Schellenberger *et al.,* (2011a). VLPs were purified from agro‐infiltrated *N. benthamiana* leaves following the same experimental procedure, except that the final discontinuous sucrose gradient was omitted. Briefly, a minimum of 350 g of leaves were ground in extraction buffer, the resulting extract was filtered, incubated with bentonite and finally clarified by centrifugation for 15 min at 1900 ***g***. VLPs were precipitated from clarified sap by adding PEG‐20 000 and sodium chloride and further processed by centrifugation on a sucrose cushion followed by a sucrose density gradient fractionation. Two millilitres of fractions was collected from which aliquots at 1 : 500, 1 : 5000 and 1 : 10 000 dilutions were processed for a semiquantitative DAS‐ELISA to identify VLP‐enriched fractions that were further pooled before final ultracentrifugation at 290 000 ***g*** for 2 h. After resuspension in HEPES 25 mm pH 8, VLPs were quantified by DAS‐ELISA (Vigne *et al*., 2013) using purified GFLV as a standard.

### SDS‐PAGE, western blot and mass spectrometry

For SDS‐PAGE analysis, 6 μg of GFLV particles equivalent from each purified sample was separated on an 8% acrylamide gel and stained with Coomassie blue using Instant Blue (Expedeon Inc., San Diego, CA). For mass spectrometry, SDS‐PAGE bands of interest were excised and proteins were destained, reduced, alkylated, trypsin‐digested overnight, chemotrypsin‐digested and finally processed for nano‐LC‐MSMS analysis on a nanoU3000 (Dionex, Thermo Fisher Scientific)‐ESI‐MicroTOFQII (Bruker, Billerica, MA). Mass spectrometry data were analysed using Mascot (Matrix Science Ltd, London, UK) and Proteinscape (Bruker). For Western blot analyses, 0.05 μg of each sample was resolved on an 8% acrylamide gel and denatured proteins were electrotransferred onto Immobilon PVDF membranes. Membranes were incubated with rabbit polyclonal anti‐GFLV antibodies at a 1 : 1000 dilution or with polyclonal anti‐TR antibodies (Evrogen) at a 1 : 5000 dilution. Proteins were detected by chemiluminescence after incubation with goat anti‐rabbit antibodies conjugated to horseradish peroxidase at a 1 : 12 500 dilution (Thermo Fisher Scientific) and with Lumi‐Light solution (Roche). Images were taken with a G : Box imaging system (Syngene, Cambridge, UK), analysed with GeneTools (Syngene) and finally processed with GIMP (www.gimp.org).

### Single‐particle epifluorescence microscopy

Purified VLPs were diluted in HEPES 25 mm pH 8 to obtain individual spots upon imaging on an inverted epifluorescence microscope Axio Observer Z1 (Zeiss) equipped with an Orca Flash4.0 camera (Hamamatsu Photonics, Massy, France) and Spectra X light engine (Lumencor, Beaverton, OR). Excitation and emission wavelength filters were 455—495 nm and 505—555 nm for EG and of 532.5—557.5 nm and 570—640 nm for TR. Images were processed as described above.

### Native agarose gel electrophoresis

Native gel electrophoresis of purified virions and VLPs was performed in 1% w/v agarose gels in 0.5X TAE buffer (20 mm TrisBase, 1.3 mm EDTA, 0.06% v/v acetic acid). For nucleic acid detection, 5 μg of virus particles or VLPs was diluted in loading buffer (10% v/v glycerol, HEPES 25 mm pH 8) supplemented with ethidium bromide (EtBr) at 0.1 μg/mL. After electrophoretic separation, the EtBr‐prestained gel was first processed for nucleic acid content using the Gel Doc system ( Bio‐Rad, Hercules, CA) equipped with a 302‐nm excitation source and a 520‐ to 640‐nm band‐pass emission filter before processing for Coomassie blue staining as mentioned previously.

For fluorescence imaging, 3 μg of purified VLPs was diluted in loading buffer and native gel electrophoresis was performed in the absence of EtBr. Imaging was performed with a G : Box imaging system (Syngene) equipped with a 450‐ to 485‐nm excitation LED module and a 510‐ to 540‐nm emission band‐pass filter for EG visualization. TR visualization was realized upon 480‐ to 540‐nm LED excitation and 590‐ to 660‐nm band‐pass emission filtering.

## Conflict of interest

No conflict of interest declared.

## Supporting information


**Figure S1** Complete amino‐acid sequence of proteins used in this study and depicted in Figure S1. Residues corresponding to CP, TagRFP, EGFP, L_1_ linker and L_2_ linker are indicated in brown, pink, green, orange and blue, respectively.
**Figure S2** Epifluorescence macroscopy images of agro‐infiltrated *N. benthamiana* leaves expressing TR, CPTR or TRCP. Scale bars: 300 μm.
**Figure S3** Sucrose gradient purification of VLPs. (a) Bright pink band after linear sucrose gradient centrifugation of TRCP VLPs (arrowheads). (b) Schematic representation of the location of virus‐ and VLP‐enriched fractions in linear sucrose gradients. The collected 2 mL fractions are numbered from 1 (top of the gradient) to 15 (bottom). RNA‐containing virions were localized measuring the A_260_ of the different fractions. VLP‐enriched fractions were identified by semiquantitative ELISA.
**Figure S4** Coverage of GFLV CP, CP, CPTR and TRCP sequences obtained by nano‐LC‐MSMS analysis of bands 1 to 8 shown in Figure S4c. Primary amino‐acid sequence of the full‐length proteins are presented. Residues belonging to CP are indicated in bold. Sequence coverage identified by NanoLC‐MSMS is indicated in red. Underlined residues correspond to first and last covered residues for each band. (a) MS analysis of band 1 (GFLV CP: 504 residues). Covered sequence starts with residue 6 and ends with residue 499. (b) MS analysis of band 2: CP with additional Met in position 1, 505 residues). Covered sequence starts with residue 2 and ends with residue 500. (c) MS analysis of bands 3, 4 and 5 (CPTR, 745 residues). Covered residues: 2‐511 (band 5), 7‐669 (band 4) and 7‐739 (band 3). (d) MS analysis of bands 6, 7 and 8 (TRCP, 744 residues). Covered residues: 123‐739 (band 8), 82‐739 (band 7) and 2‐739 (band 6). Untreated MS analysis results can be provided upon request.
