# Supplementary material for: Display of whole proteins on inner and outer surfaces of grapevine fanleaf virus‐like particles
Source: Plant Biotechnol J. 2016 Jul 29;14(12):2288–99. doi: 10.1111/pbi.12582 (PMC5103221; doi:10.1111/pbi.12582)
Supplement: Supplementary file 1 — Figure S1 Complete amino‐acid sequence of proteins used in this study and depicted in Figure S1. Residues corresponding to CP, TagRFP, EGFP, L1 linker and L2 linker are indicated in brown, pink, green, orange and blue, respectively. Figure S2 Epifluorescence macroscopy images of agro‐infiltrated N. benthamiana leaves expressing TR, CPTR or TRCP. Scale bars: 300 μm. Figure S3 Sucrose gradient purification of VLPs. (a) Bright pink band after linear sucrose gradient centrifugation of TRCP VLPs (arrowheads). (b) Schematic representation of the location of virus‐ and VLP‐enriched fractions in linear sucrose gradients. The collected 2 mL fractions are numbered from 1 (top of the gradient) to 15 (bottom). RNA‐containing virions were localized measuring the A260 of the different fractions. VLP‐enriched fractions were identified by semiquantitative ELISA. Figure S4 Coverage of GFLV CP, CP, CPTR and TRCP sequences obtained by nano‐LC‐MSMS analysis of bands 1 to 8 shown in Figure S4c. Primary amino‐acid sequence of the full‐length proteins are presented. Residues belonging to CP are indicated in bold. Sequence coverage identified by NanoLC‐MSMS is indicated in red. Underlined residues correspond to first and last covered residues for each band. (a) MS analysis of band 1 (GFLV CP: 504 residues). Covered sequence starts with residue 6 and ends with residue 499. (b) MS analysis of band 2: CP with additional Met in position 1, 505 residues). Covered sequence starts with residue 2 and ends with residue 500. (c) MS analysis of bands 3, 4 and 5 (CPTR, 745 residues). Covered residues: 2‐511 (band 5), 7‐669 (band 4) and 7‐739 (band 3). (d) MS analysis of bands 6, 7 and 8 (TRCP, 744 residues). Covered residues: 123‐739 (band 8), 82‐739 (band 7) and 2‐739 (band 6). Untreated MS analysis results can be provided upon request. [file PBI-14-2288-s001.docx]

**Supporting Information**

**Figure 1 SuppInfo** Complete amino-acid sequence of proteins used in this study and depicted in Figure 1. Residues corresponding to CP, TagRFP, EGFP, L_1_ linker and L_2_ linker are indicated in brown, pink, green, orange and blue, respectively.

**Figure 2 SuppInfo** Epifluorescence macroscopy images of agro-infiltrated *N. benthamiana* leaves expressing TR, CPTR or TRCP. Scale bars: 300 µm.

**Figure 3 SuppInfo** Sucrose gradient purification of VLPs. (a) Bright pink band after linear sucrose gradient centrifugation of TRCP VLPs (arrowheads). (b) Schematic representation of the location of virus- and VLP-enriched fractions in linear sucrose gradients. The collected 2 mL fractions are numbered from 1 (top of the gradient) to 15 (bottom). RNA-containing virions were localized measuring the A_260_ of the different fractions. VLP-enriched fractions were identified by semi-quantitative ELISA.

**Figure 4 SuppInfo** Coverage of GFLV CP, CP, CPTR and TRCP sequences obtained by NanoLC-MSMS analysis of bands 1 to 8 shown in Figure 4c. Primary amino-acid sequence of the full-length proteins are presented. Residues belonging to CP are indicated in bold. Sequence coverage identified by NanoLC-MSMS is indicated in red. Underlined residues correspond to first and last covered residues for each band. (a) MS analysis of band 1 (GFLV CP: 504 residues). Covered sequence starts with residue 6 and ends with residue 499. (b) MS analysis of band 2: CP with additional Met in position 1, 505 residues). Covered sequence starts with residue 2 and ends with residue 500. (c) MS analysis of bands 3, 4 and 5 (CPTR, 745 residues). Covered residues: 2-511 (band 5), 7-669 (band 4) and 7-739 (band 3). (d) MS analysis of bands 6, 7 and 8 (TRCP, 744 residues). Covered residues: 123-739 (band 8), 82-739 (band 7) and 2-739 (band 6). Untreated MS analysis results can be provided upon request.
